# Alopecia Areata Universalis Precipitated by SARS-CoV-2 Vaccine: A Case Report and Narrative Review

**DOI:** 10.7759/cureus.27953

**Published:** 2022-08-12

**Authors:** Hani Abdalla, Ebrahim Ebrahim

**Affiliations:** 1 Family Medicine, Primary Health Care Corporation, Doha, QAT; 2 Medical Education/Family Medicine, Hamad Medical Corporation, Doha, QAT

**Keywords:** vaccine safety, sars-cov2, covid-19, hair loss, alopecia areata universalis, alopecia areata

## Abstract

Alopecia areata (AA) is a patchy autoimmune nonscarring hair loss. Various pathophysiological explanations are described with immune dysregulation being the most well established. In this report, we describe a 63-year-old lady with AA recurrence, in the form of AA universalis, after 32 years of remission, following administration of severe acute respiratory syndrome coronavirus 2 (SARS-CoV-2) vaccine. We also briefly reviewed published cases with similar presentations after receiving the SARS-CoV-2 vaccine.

## Introduction

Hair loss is a common presentation in primary care. Among various causes of hair loss, alopecia areata (AA) represents 18.2% of the causes [1] and has a lifetime risk of approximately 2% [2,3]. AA is a patchy nonscarring alopecia with underlying autoimmunity against hair follicles with resultant dystrophy of the hair follicle at the anagen phase of growth [3,4]. Severe forms of AA include alopecia totalis (all scalp hair) and universalis (entire body). Despite multiple underlying pathophysiological mechanisms, there are two well-established primary explanations. The immune dysregulation with loss of immune privilege of hair follicles and genetic predisposition [3,5]. Other factors include infections, drugs, and vaccines with consequent immune dysregulation and development of AA.

Wise, Kiminyo, and Salive's 1997 study was among the earliest reports of hair loss after routine immunizations in their case series [6]. It is suggested that vaccines via antigen presentation, cytokine production, epitope spreading, polyclonal activation of B cells, and other mechanisms of anti-infectious immune response and autoreactivity potentially trigger autoimmunity [7]. Regarding severe acute respiratory syndrome coronavirus 2 (SARS-CoV-2) vaccines, there is a theoretical risk of inducing autoimmunity and a number of reports of different autoimmune sequela [8]; however, most frequently used messenger ribonucleic acid (mRNA) vaccines have excluded patients with a history of autoimmune conditions from their clinical trials. The US Food and Drug Administration has raised the concern of possible precipitation of rheumatoid arthritis after SARS-CoV-2 vaccination [9]. Pfizer vaccine developers have included a list of rare complications, including myocarditis and skin reactions, in the information leaflet, but AA was not listed.

In addition to the morbidity burden of AA, it is distressing to affected patients and is associated with major psychosocial sequelae and reduced quality of life [10]. This report presents a patient with alopecia universalis recurrence precipitated by the SARS-CoV-2 vaccine.

## Case presentation

A 63-year-old lady previously known to our primary care service with hypothyroidism, prediabetes, and thalassemia trait presented to our office with a new complaint of extensive hair loss. Her condition started with patchy hair loss within two weeks from the first dose of the SARS-CoV-2 (Pfizer mRNA) vaccine. Hair loss was progressive until the second dose which resulted in further rapid loss with consequent complete hair loss of entire body hair in six weeks from the first dose. There were no other active complaints and no history of using hair care products prior to the event.

The patient’s hypothyroidism was diagnosed 17 years ago and is adequately treated with levothyroxine. She was diagnosed with prediabetes six years ago based on elevated fasting glucose and hemoglobin A1C readings and is using metformin XR daily. She also reported seasonal symptoms consistent with allergic rhinitis. She denied symptoms and history of vitiligo, psoriasis, atopic dermatitis, systemic lupus erythematosus, or other dermatologic or rheumatological illnesses. Aside from vitamin D3 supplementation, her other medications are Pantogar® capsules, methylcobalamin, and ferrous gluconate, all of which are not prescribed by our service. The patient has a penicillin allergy. There was no recent surgical insult and her surgical records show tonsillectomy and a cesarean section at a young age. There was a past distant history of blood transfusion for an obstetric indication. Her obstetric history included two fetal losses at six and two months gestation, respectively.

Our patient is married with four living children and has been postmenopausal for 16 years now. She has never smoked or consumed alcohol. Her family history is unremarkable except for a daughter with subclinical hypothyroidism and positive anti-thyroid peroxidase (TPO) and another daughter with subfertility attributed by her treating physician to an autoimmune etiology, no family history of AA was reported. The patient’s immunization records show both SARS-CoV-2 received in October 2021, pneumococcal polysaccharide vaccine 23 and influenza vaccines in 2020, and pneumococcal conjugate vaccine 13 and tetanus, diphtheria, and acellular pertussis vaccine in 2019. The patient declined the latest seasonal flu vaccine and expressed her disinterest in the third SARS-CoV-2 booster. The patient has not contracted coronavirus disease 2019 (COVID-19) infection during the pandemic and has always tested negative in screening during contact tracing or for travel purposes.

Enquiry about any similar history of hair loss revealed an episode of patchy hair loss 32 years ago with a formal diagnosis of alopecia areata which wasn’t volunteered until meticulous questioning.

Physical examination shows a slightly overweight patient with complete loss of scalp, facial, and body hair. Skin inspection shows no other findings and an unremarkable general examination. Thorough cardiovascular, pulmonary, abdominal, neurological, and musculoskeletal exams are unremarkable. Her laboratory evaluation shows a normal complete blood picture, ferritin, vitamin D, B12, thyroid function tests, C-reactive protein, erythrocyte sedimentation rate, and complete metabolic panel. She tested negative for rheumatic factor, anti-citrullinated peptide, anti-nuclear antibody, and viral markers with evidence of effective hepatitis B vaccination. The patient was offered referral to dermatology, however, she preferred to follow with a dermatologist privately.

## Discussion

Patient’s evaluation leads to the diagnosis of AA recurrence in the form of alopecia areata universalis (AAU) precipitated by SARS-CoV-2 vaccine and is reported as the first local case (Qatar). Vaccines (e.g. herpes zoster, hepatitis B, human papillomavirus, etc.) have been traditionally linked to AA occurrence and recurrence [11,12]. In line with the contemporary pandemic of SARS-CoV-2 and with the increasing number of people receiving one of its various vaccines, there has been a growing quantity of published reports highlighting a possible link to AA occurrence or recurrence [13-18]. Most of these reports described the condition following BNT162b2 mRNA Pfizer, mRNA-1273 Moderna, and AZD1222/ChAdOx1 Oxford AstraZeneca vaccines. Despite variation in the mechanisms of these two groups of vaccines, both share the goal of inducing antibody humoral response against viral particles [19]. In patients with background susceptibility and dysregulated immunity, response to these vaccines may differ and trigger their autoimmunity.

Severity of AA ranged from a patchy disease of single to multiple patches, to an extensive AAT or AAU [13-18]. In our patient patchy AA occurred initially then progressed to complete AAU within six weeks with major psychosocial distress. The onset of flare has shown great temporality with the vaccine administration and this has been also seen in other reports as shown in Table 1. Despite this temporality, clear and quantifiable association cannot be established without further studies of appropriate design.

**Table 1:** Case profile of published reports PAA: Patchy alopecia areata, AAT: Alopecia areata totalis, AAU: Alopecia areata universalis, HBV: hepatitis B virus, NA: Not applicable

Authors	Number of cases / Location	Vaccine type	Age	Sex	Time to diagnosis from 1^st^ dose	AA type	PMHx	Other remarks
Scollan ME, et al. [13]	9 United States	6 Pfizer 3 Moderna	15-62 years	5 females 4 males	1-19 weeks Average 7 weeks	1 AAT 2 AAU 6 PAA	1 Chronic HBV 1 Hashimoto thyroiditis 2 Elevated thyroid Antibodies 1 Joint pain on hydroxychloroquine 2 previous AA 2 NA	Moderna appeared to induce the flare later that Pfizer vaccine
Gallo G, et al. [14]	1 Italy	Pfizer	31 years	Male	3 weeks (1 day from 2^nd^ dose)	PAA	Negative past medical history	None
Bardazzi F, et al. [15]	3 Italy	2 Pfizer 1 Moderna	28-59 years	2 females 1 male	2-3 weeks	1 AAT 2 PAA	Chronic recurrent AA in 1 patient and single PAA as a child in another.	Poor response to local and systemic corticosteriods
May Lee M, et al. [16]	1 Italy	Pfizer	80 years	Male	1 week	AAT	Negative past medical history	AAT by 8 weeks No response to topical immunotherapy & Minoxidil.
Essam R, et al. [17]	1 Egypt	Oxford Astrazenca	32 years	Female	< 1 week	PAA	One previous mild attack PAA COVID-19 infection a year ago	Probably the first described case
Rossi A, et al. [18]	3 Italy	2 Oxford AstraZenca 1 Pfizer	29, 59, & 76 years	3 females	2-3 weeks	All PAA	One patient had PAA 4 years before current episode. One patient had autoimmune thyroiditis and 2 episodes of PAA One patient had Ophiasis 2 years before current episode.	Follow-up scheduled, disease outcome not yet published.

Since many other factors have been known to provoke AA, we have been meticulous in history taking to detect other factors that may have coexisted simultaneously. In fact, the recall of the previous episode of AA was not volunteered until the second visit. Most of available literature reported previous history of AA either remote or recent, however, May Lee et al., 2021, and Gallo et al., 2021, have reported cases for the first time [14,16].

Another risk factor of clinical relevance in our patient was her Hashimoto’s thyroiditis. This is parallel to existing literature reporting autoimmune thyroiditis as a widespread and significant association with AA [11]. Table 1 also highlights Hashimoto’s thyroiditis and thyroid antibodies as the leading conditions in past history review. Although seasonal increase of AA flares is discussed in literature with peak season lasting from September to April, the higher incidence of respiratory infections in these months could be a major confounder [20]. In our case, this seasonal risk is less likely with such a short interval between the vaccine and the AA flare.

The progression of diseases appears to be unpredictable and prognosis is variable according to severity with many limited diseases end into remission and extensive AAT or AAU carrying poor prognosis. Therefore, counselling patients regarding expected course and prognosis is challenging.

## Conclusions

Knowledge about SARS-CoV-2 vaccine safety and benefits is evolving to support decision-making about use of these vaccines. Although benefits of these vaccines greatly overweight risks associated with acquisition of infection, the benefit-risk balance should be communicated to patients. There is currently a lack of clear-cut recommendations about screening for autoimmunity in patients receiving SARS-CoV-2 vaccines and autoimmunity in this context is multifactorial with multiple modifiers. Due to the growing reports of autoimmunity flares including AA, healthcare providers should remember to enquire about personal and/or family history of autoimmunity. This would allow for proper patient-centered counselling and enable patients to take informed health decisions in their best interest.
